# Liver Metastases and Histological Growth Patterns: Biological Behavior and Potential Clinical Implications—Another Path to Individualized Medicine?

**DOI:** 10.1155/2019/6280347

**Published:** 2019-02-25

**Authors:** Rui Caetano Oliveira, Henrique Alexandrino, Maria Augusta Cipriano, José Guilherme Tralhão

**Affiliations:** ^1^Pathology Department, Centro Hospitalar e Univeristário de Coimbra, 3000-075, Coimbra, Portugal; ^2^Biophysics Institute, Faculty of Medicine, University of Coimbra, 3000-548 Coimbra, Portugal; ^3^Coimbra Institute for Clinical and Biomedical Research (iCBR) area of Environment Genetics and Oncobiology (CIMAGO), Faculty of Medicine, University of Coimbra, 3000-548 Coimbra, Portugal; ^4^Surgery Department, Centro Hospitalar e Universitário de Coimbra, 3000-075, Coimbra, Portugal; ^5^Faculty of Medicine, University of Coimbra, 3000-548, Coimbra, Portugal

## Abstract

Colorectal cancer is a major health burden and despite the recent advances in healthcare and screening programs, a great percentage of patients already present metastases once their disease is found. In those cases, liver surgery has an essential role, but even with neoadjuvant chemotherapy there is a high rate of intrahepatic recurrence. New prognostic factors are needed in order to decide the best surgical approach considering the biological behavior of the tumors in order to tailor the used therapies, moving towards individualized medicine/treatment. However, the majority of the factors described in literature are expensive, time consuming, and difficult to apply on a daily basis. Histological growth patterns have emerged over the past few years as a reproducible characteristic, an easy to apply one, and with very low costs since it only needs the standard Haematoxylin and Eosin stained slides of observation. In this article, we provide a review of the histological growth patterns of liver metastases and their prognostic significance, biological meaning, and therapeutic importance.

## 1. Introduction

The liver is a common site for metastatic dissemination and in some regions of the globe, namely, Europe, secondary liver tumors are far more common than primary [1] ones. Regarding metastatic disease the adenocarcinomas are predominant, and colorectal carcinoma is the most prevalent place of origin, having a high mortality [2].

In the past few years major advances regarding treatment strategies of colorectal cancer liver metastases (CRCLM), such as more effective chemotherapy regimens, portal vein embolization and staged hepatectomies (including Associating Liver Partition and Portal Vein Ligation for Staged Hepatectomy, ALPSS), and one-stage ultrasound-guided parenchymal preserving resections, all have contributed to extend the limits of oncological resectability [3, 4]. In selected cases, liver transplantation has also been used successfully [5]. However, regardless of the curative intent, intrahepatic recurrence has been reported in more than 50% of the cases, even with adjuvant chemotherapy [6]. Several retrospective studies have identified these patients' cohorts with poor prognostic factors such as tumor size, number of lesions, and tumor progression after chemotherapy or shorter interval from primary tumor surgery [7]. Nevertheless, none of these are absolute contraindications for hepatic surgery and do not represent the tumor-host interaction that will be required for individualized medicine/treatment. More aggressive hepatic surgeries did not show improved survival [8] rates and neoadjuvant chemotherapy is associated with increased postoperative morbidity [9]. New prognostic and biomarkers are thus of paramount importance.

## 2. The Detailed Study of CRCLM and Its Importance

**Figure 1:**
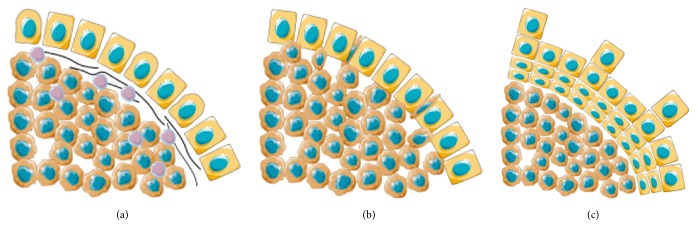
Schematic representation of the Growth Patterns of Colorectal Liver Metastases, adapted from Temido MJ (2018) Clinical and Pathological Factors of Prognosis after Hepatectomy for Gastric Cancer Liver Metastases. Is desmoplastic growth the key to longer survival? Master Thesis in Medicine, with permission. (a) Desmoplastic Growth Pattern: the tumor is separated from the liver parenchyma by a band of fibrous tissue, which contains tumor infiltrating lymphocytes; (b) Replacement Growth Pattern: the tumor permeates between the liver hepatocytes, without disruption of the normal architecture; (c) Pushing Growth Pattern: the tumor expands and compresses the surrounding hepatocytes.

**Figure 2:**
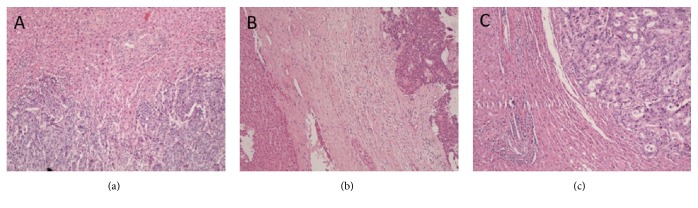
Haematoxylin & Eosin (H&E) evaluation of the Colorectal Liver Metastasis Growth Patterns, adapted from Falcão, D. et al. Histopathologic patterns as markers of prognosis in patients undergoing hepatectomy for colorectal cancer liver metastases: Pushing growth as an independent risk factor for decreased survival. Eur. J. Surg. Oncol. (2018). doi:10.1016/j.ejso.2018.03.02, with permission. (a) Replacement Growth Pattern: the tumor permeates between the liver hepatocytes, without disruption of the normal architecture, H&E 100x; (b) Desmoplastic Growth Pattern: the tumor is separated from the liver parenchyma by a band of fibrous tissue, which contains tumor infiltrating lymphocytes, H&E 100x; (c) Pushing Growth Pattern: the tumor expands and compresses the surrounding hepatocytes, H&E 100x.

Recently, histological growth patterns (GP) have been identified as a practical and reproducible factor of prognosis, easily assessed by Haematoxylin and Eosin (H&E) stained slides by an experienced pathologist. They are defined as expansive (when tumor growth compresses the hepatocytes), desmoplastic (with presence of fibrous tissue in the periphery of the tumor), and replacement (when tumor infiltrates the hepatocytes without architectural changes) (Figures 1 and 2). Our group and others have demonstrated that a pushing growth pattern is related to a worse prognosis [10] while a desmoplastic growth pattern is associated with a more favorable outcome [11]. What is particularly interesting about the GP is that the information is readily available on routine H&E pathological examination and does not require lengthy or expensive ancillary studies. This may be particularly important in low-resource settings.

The correct analysis and consequent accurate classification of the CRCLM growth pattern implies a detailed gross examination with adequate sampling with at least one sample per tumor centimeter, similar to the sampling used for tumor regression grade assessment, for all the lesions [12, 13]; in our institution we perform full sampling of all lesions with size inferior to 3cm.

Other studies have attempted to identify the characteristics that allow for a better or worse prognosis for overall survival such as tumor thickness at the tumor-normal interface [14, 15] and study of the tumor infiltrating lymphocytes and its composition [16, 17]. However, this type of evaluation is complicated, time consuming, and requires special software.

## 3. Why Are There Different Growth Patterns?

The reason for this behavior has not been fully understood yet. However, it can represent the complexity of tumor/host interactions, with the pushing pattern described by some authors as more angiogenic [18] and the desmoplastic as an inflammatory response of the host; it is also probably related to the response to chemotherapy [19].

The thick band of stroma present in the desmoplastic growth pattern, enriched with collagen, may act as an obstacle to tumor expansion, representing an improved host response with dense lymphocytic infiltration, increase in collagen type IV and integrin blockade, reducing the infiltration of nontumoral parenchyma, therefore demonstrating a more favorable characteristic [10, 20].

The pushing pattern displays biologic properties with increased levels of endothelial cell proliferation fraction [18, 21] and upregulation of vascular factors, such as basic fibroblast growth factor (bFGF) and vascular endothelial growth factor (VEGF) [22], sometimes in a similar mode as in the primary colorectal cancer [23]. The pushing pattern is also characterized by a hypoxic environment, a well-known factor of aggressiveness, and resistance to therapy [24, 25].

The information gained by studying the molecular mechanisms underlying the complex tumor/host interactions associated with the distinct GPs may aid in the selection of new therapeutic targets. Growth receptor blockade (with anti-EGFR antibodies) and antiangiogenic agents (with anti-VEGF antibodies) have already demonstrated excellent results in the treatment of advanced metastatic disease.

Several studies have addressed the relation of GPs and angiogenesis capacity, and the recent discovery mechanism of vessel cooption vascularization of tumors has explained a possible resistance to antiangiogenic agents. This led to the suggestion of a combined therapeutic as possible approach and linked this biological behavior to a specific GP [26].

Other mechanisms of tumoral survival, such as evading the host immune response, epithelial-mesenchymal transition, or hypoxia-resisting factors, may serve as targets for molecular therapies in the near future, namely, immunotherapy [27, 28] and cell cycle inhibitors [29, 30].

By reflecting the tumor-host interaction, the GP of liver metastases can influence overall and disease-free survival. Although GP could influence patient management, being a histopathological characteristic, the GP can only be known after surgical resection. However, imaging techniques could potentially detect different types of growth patterns before surgery.

Due to the simplicity of this biological characteristic, other studies have assessed this biological behavior in liver metastases of nonintestinal carcinomas, such as breast cancer [31] and uveal melanoma [32], but in these cases there is a predominance of the replacement pattern and consequently a worse prognosis.

## 4. Can we Predict the Growth Pattern before Surgery?

The different prognoses of the colorectal cancer liver metastases (CRCLM) may represent a new prognostic characteristic that may be related to the primary tumor properties and may be predicted by preoperative imaging, allowing individualized patient care.

The radiological response pattern to chemotherapy, particularly with antiangiogenic drugs, has been previously reported as having implications in the prognosis. In a cohort of 209 patients with CRCLM undergoing hepatectomy after neoadjuvant chemotherapy, the presence of a sharply defined border on preoperative computed tomography (CT) was associated with improved overall and disease-free survival [33]. However, the tumor-hepatic tissue interface was not assessed in this study and thus it remains to be answered whether this radiologic pattern corresponded to a distinct histological GP.

More accurate in tissue analysis than CT, Magnetic Resonance imaging (MRI) could provide important answers in this regard, especially with the use of hepatospecific contrast agents. In fact, the relative tumor enhancement by gadoxetic acid (a contrast agent used in hepatobiliary imaging) can accurately predict the response to chemotherapy in treatment-naïve patients with CRCLM [34]. Moreover, quantitative texture analysis in MRI, using radiomics, can also potentially detect microstructural changes in the liver parenchyma. In one study using an animal model of CRCLM, micrometastases were detected by radiomics before histopathological expression [35]. Hopefully this can also be used in the analysis of tumor-host tissue interface. Further studies will still have to be conducted.

One may raise the question about patients with several metastases; in our study [11], the majority of the CRCLM presented the same GP. It would be a very interesting study to see if in patients submitted to second and third hepatectomy the GP remained the same; some studies have assessed this and it seems that there is maintenance of GP [36]. This would allow better selection of patients for second and third hepatectomy.

## 5. Implications of CRCLM Growth Pattern in Treatment

Advances in imaging could detect distinct GPs, thus impacting patient management. This could lead to a tailoring of the therapy, both in the choice and duration of chemotherapy and in the use of resection and other locoregional techniques.

In fact, there are two distinct currents of thought in the scientific literature regarding the surgical management of CRCLM [4]: on the one hand, proponents of radical, R0 resections, even if requiring major or extended hepatectomies associated with parenchyma-modulating strategies, such as portal vein embolization or the Associating Liver Partition and Portal Vein Ligation for Staged Hepatectomy (ALLPS) [37, 38] and, on the other hand, proponents of a parenchymal-sparing, radical but conservative approach, whereby metastases are resected leaving the liver's vascular and biliary structures intact, even if at the cost of R1 resection [39]. However, colorectal cancer liver metastasis is a heterogeneous disease and different patients might present different growth patterns, possibly representing distinct tumor-host interactions. We speculate whether a R1 resection might still be curative in a patient with desmoplastic growth pattern, but not in a patient with pushing growth pattern. This, however, remains unproven.

Moreover, knowledge of the GP could also influence the choice and duration of chemotherapy regimens. Although beneficial, preoperative chemotherapy in CRCLM can cause hepatotoxicity and increase postoperative morbidity [9, 40–42] Although evidence for this is scant, in our previous study we found that patients treated with a combination of oxaliplatin and 5-fluoruracil (FOLFOX) were more likely to present a pushing GP on the pathological analysis of the resected specimen [11], in which way this information aid in the choice of chemotherapy is still unknown.

Histological GPs are indeed a powerful tool, but in the past, they were described using several designations, thus raising barriers for worldwide harmonisation [43]. The recent development of a consensus should provide this parameter with enough strength and reproducibility for daily clinical use [44].

Concerning the surgical approach, literature is not unanimous regarding the perfect approach to synchronous CRCLM: some advocate the colorectal surgery first [45], others advocate a liver first approach [46], and finally are those who perform a synchronous resection [47, 48]. Nevertheless, no single strategy gains unanimity among surgeons [45].

The possibility of predicting the GP of CRCLM in preoperatory evaluation may allow for an individualized treatment algorithm for each patient. Particular imaging features of the metastatic disease could also expand this information. In this way, neoadjuvant chemotherapy, known to be more effective on the secondary tumor than on the primary [49] one, could be adequately selected, in both the choice of the agents and the duration. In addition, timing and extent of surgical resection could also be selected according to the risk of intrahepatic recurrence.

## 6. Conclusion

We hope that further investigation into GPs can help clinicians to choose therapies in a multimodal perspective, not only in cases of CRCLM but also in other indications, such as gastric or breast cancer liver metastases. The consistent report of the CRCLM GP in pathology reports according to the correct consensus [44] should be a powerful and consistent characteristic for behavior prediction. In the near future, we envision that imaging may provide important answers regarding GPs, and this knowledge may help the selection of the right therapy for each patient.
